# Noncoding RNAs in disease

**DOI:** 10.1002/1873-3468.13182

**Published:** 2018-07-20

**Authors:** Evangelia Lekka, Jonathan Hall

**Affiliations:** ^1^ Department of Chemistry and Applied Biosciences Institute of Pharmaceutical Sciences ETH Zürich Switzerland

**Keywords:** long noncoding RNA, microRNA, noncoding RNA

## Abstract

Noncoding RNAs are emerging as potent and multifunctional regulators in all biological processes. In parallel, a rapidly growing number of studies has unravelled associations between aberrant noncoding RNA expression and human diseases. These associations have been extensively reviewed, often with the focus on a particular microRNA (miRNA) (family) or a selected disease/pathology. In this Mini‐Review, we highlight a selection of studies in order to demonstrate the wide‐scale involvement of miRNAs and long noncoding RNAs in the pathophysiology of three types of diseases: cancer, cardiovascular and neurological disorders. This research is opening new avenues to novel therapeutic approaches.

## Abbreviations

**AD**, Alzheimer's disease

**ALS**, amyotrophic lateral sclerosis

**APP**, amyloid precursor protein

**BDNF**, brain‐derived neurotrophic factor

**CARL**, cardiac apoptosis‐related lncRNA

**CLL**, chronic lymphocytic leukaemia

**CRP**, C‐reactive protein

**EC**, endothelial cell

**FTLD**, frontotemporal lobar degeneration

**HCC**, hepatocellular carcinoma

**HD**, Huntington's disease

**lncRNAs**, long noncoding RNAs

**miRNA**, microRNA

**MPTP**, 1‐methyl‐4‐phenyl‐1,2,3,6‐tetrahydropyridine

**NATs**, natural antisense transcripts

**ncRNA**, noncoding RNA

**NSCLC**, nonsmall cell lung cancer

**PD**, Parkinson's disease

**SMA**, spinal muscular atrophy

Completion of the Human Genome Project has revealed that protein‐coding genes comprise only about 1.5% of the human genome. In fact, two large‐scale consortia, the Encyclopedia of DNA elements (ENCODE) and the Functional Annotation of the Mammalian Genome (FANTOM) have shown that the majority of genome is transcribed and produces a wide spectrum of noncoding RNA species (ncRNAs) 1, 2, 3, 4. Consequently, it is now believed that the degree of complexity of a species correlates better with the number of ncRNAs than with the number of protein‐coding genes 5. Furthermore, the availability of this data has shown that mutations within the noncoding genome are major determinants of human diseases, for example cancer 6.

Noncoding RNAs can be classified, according to their size: short RNAs are < 200 nucleotides (nts) in length and include small interfering RNAs (siRNAs), piwi‐interacting RNAs (piRNAs) and microRNAs (miRNAs) 7, 8; Long noncoding RNAs (lncRNAs) are longer than 200 nts and may comprise thousands of nucleotides 9. Thanks to their major contributions in so many cellular processes, the study of ncRNAs has evolved into a rather inspiring scientific field.

The discovery of miRNAs dates back to 1993, when two laboratories independently reported that a small noncoding RNA transcript lin‐4 from *Caenorhabditis elegans* regulates lin‐14 through its 3′ untranslated region (3′UTR) 10, 11. At the time of their discovery, it was unclear whether miRNAs were an odd RNA species or ‘emissaries from an unexplored RNA world’ 12. The intense research which followed showed that miRNAs are key regulatory elements of gene expression and essential mediators in a wide range of cellular processes in both health and disease.

**Figure 1:**
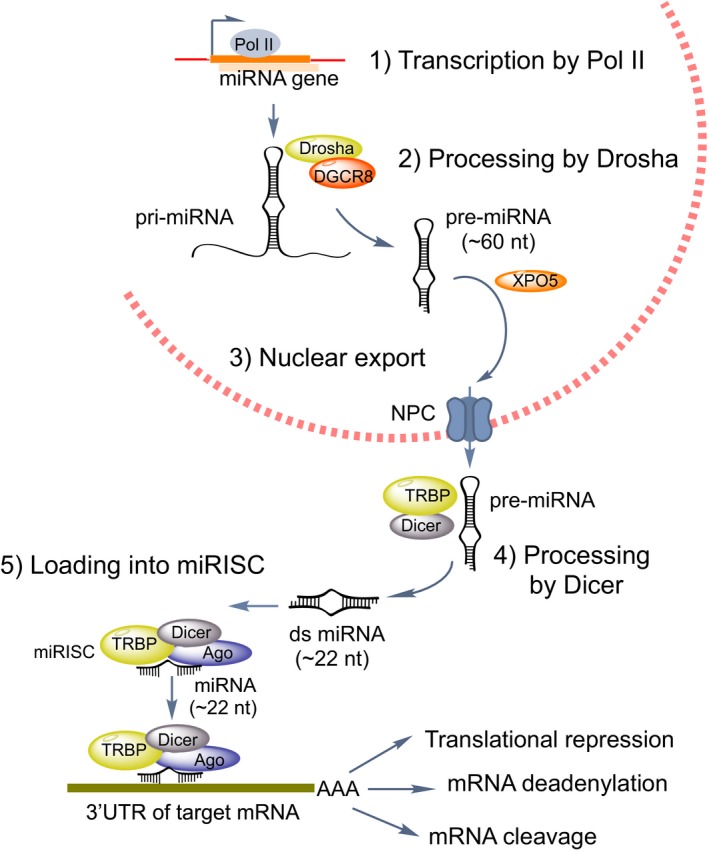
The individual steps of miRNA biogenesis.

The biogenesis of miRNAs (Fig. 1) has been reviewed in detail elsewhere 7. Briefly, miRNAs are expressed as mono‐cistronic primary transcripts or as clusters from polycistronic primary transcripts. MiRNA genes are located in defined transcriptional units or in intergenic regions. Intragenic miRNAs can be found in introns or exons of coding genes (host genes) in the sense orientation. Intragenic miRNAs and their host genes are frequently co‐ordinately expressed, since they share the same promoter 13. Their transcription is driven by RNA Polymerase II (Pol II) producing primary transcripts – called pri‐miRNAs – which are 5′‐capped, spliced and polyadenylated 14. The pri‐miRNA is cleaved at the stem of the hairpin structure by the RNaseII endonuclease III Drosha, together with DGCR8/Pasha proteins resulting in the release of a 60–70 nt hairpin structure, known as the precursor‐miRNA (pre‐miRNA). Pre‐miRNAs are then transported to the cytoplasm by the RanGTP‐dependent nuclear transporter exportin‐5 (XPO5), where they are subsequently processed by an endonuclease cytoplasmic RNase III enzyme Dicer to yield the mature miRNA of 18–25 nt length embedded in an imperfect duplex which is incorporated into the RNA‐Induced Silencing Complex (RISC), together with an Argonaute (Ago) core protein component. One strand of the miRNA duplex (the ‘passenger’ strand) is removed, whereas the other remains bound to Ago as the mature miRNA ‘guide’ strand responsible for guiding RISC to the target mRNAs 8.

MiRNAs attenuate the expression of their target genes by hybridizing, either completely or partially, to complementary binding sites located in the 3ʹUTR of target mRNAs. This leads to mRNA degradation and/or translational inhibition 15. In mammals, miRNAs promote mRNA destabilization, by recruiting the CCR4‐NOT deadenylase complex onto target mRNAs leading to deadenylation. Additionally, miRNAs can mediate translational repression, through various mechanisms, including the recruitment of downstream translational repressors 16.

Bioinformatic predictions suggest that human miRNAs regulate over 60% of transcripts. Given that a single miRNA can regulate the expression of over one hundred mRNAs 8, and each mRNA can be targeted by several miRNAs, miRNAs are highly versatile players in regulatory networks. Furthermore, RNAs containing binding sites for a certain miRNA can attenuate their activity by acting as ‘decoys’ or ‘sponges’, thereby influencing the expression of its other target RNAs 17. The roles of miRNAs also extend beyond suppression of gene expression, as they have also been reported to induce translation of targeted mRNAs 18.

Long noncoding RNAs are a large and diverse class of transcribed RNAs that lack functional open reading frames, though exceptions have been described 19. They are transcribed by RNA Pol II, and are 5′‐capped, spliced and polyadenylated 20. LncRNAs can fold into a variety of secondary structures which facilitate their interactions with DNA, RNA and proteins 21. LncRNAs can be divided into different classes based upon their genomic location: long intergenic noncoding RNAs (lincRNAs) genes are located between coding or noncoding genes. Some lncRNAs are located in the introns of protein‐coding genes. Natural antisense transcripts (NATs) are transcribed from the opposite strand of a coding gene but their transcription start site resides downstream relative to that of the host gene, and these transcripts often overlap with the sequence of the corresponding mRNA.

**Figure 2:**
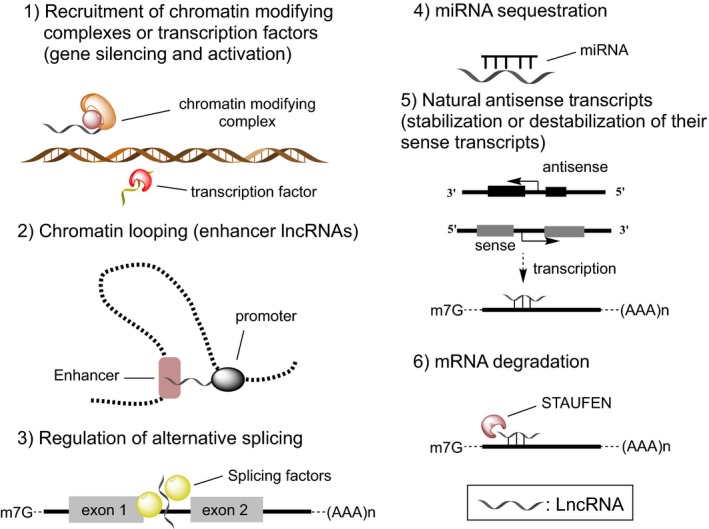
LncRNAs show a wide variety of functions.

Long noncoding RNAs function through heterogeneous mechanisms (Fig. 2), conferring additional layers of regulation upon gene expression during for example cell proliferation, cell cycle, metabolism, apoptosis, differentiation and maintenance of pluripotency 22. They also participate in chromatin modification and structure by acting as molecular scaffolds, interacting with components of the epigenetic machinery, such as histone‐modifying enzymes and DNA methyltransferases, and thus mediating their recruitment to DNA loci 23. Additionally, lncRNAs can impact the transcription of other genes, by promoting or preventing the binding of transcription factors and transcriptional mediators to promoters 24, 25. LncRNAs are involved in the regulation of RNA processing, such as RNA splicing 26, or mRNA decay 27.

Certain lncRNAs have enhancer‐like properties. Orom *et al*. 28 demonstrated that depletion of a lncRNA at multiple sites of the human genome leads to a specific decrease in the expression of neighbouring protein‐coding genes. Enhancer‐derived lncRNAs (eRNAs) are described to control contacts between enhancers and the cognate promoter through chromosome looping. Activating ncRNAs (ncRNA‐a) mediate DNA looping and chromatin remodelling *via* the Mediator complex to establish a stable transcription initiation process 29. LncRNAs can additionally function as decoy RNAs, by binding and titrating away miRNAs 17. These lncRNAs may harbour sites complementary to miRNA sequences thereby sequestering them and preventing them from binding to their targets.

A special class of lncRNAs are the antisense lncRNAs (NATs) that are transcribed from the opposite strand of a protein‐coding gene locus 30. NATs have either positive 31 or negative effects on the levels of its corresponding sense transcript 32. For example BACE1‐AS is transcribed from the β‐secretase‐1 (BACE1) gene in antisense direction: it binds to BACE1 mRNA and protects it from miRNA‐mediated degradation 31. Brain‐derived neurotrophic factor (BDNF), on the other hand, is normally repressed by a conserved noncoding antisense RNA transcript, BDNF‐AS, by recruiting the enhancer of zeste homolog 2 (EZH2) and polycomb repressive complex 2 (PRC2) to the BDNF promoter region 32. Finally, lncRNAs can interact with proteins to modulate protein function, regulate protein – protein/DNA/RNA interactions, or direct their localization within cellular compartments 33.

## MiRNAs and long noncoding RNAs in disease

In the sections below, we highlight a nonexhaustive selection of examples that demonstrate the wide‐scale involvement of miRNAs and lncRNAs in the pathophysiology of cancer, cardiovascular and neurological disorders.

### MiRNAs and long noncoding RNAs in cancer

MiRNAs play various roles in processes underlying human malignancies, including sustaining proliferation, resistance to apoptosis, angiogenesis, invasion and metastasis. Altered miRNA expression patterns found in cancer have been attributed to genomic abnormalities (deletions, amplifications or mutations) 34, epigenetic modifications 35, dysregulated transcription factors 36 and dysregulation of RNA‐binding proteins (RBPs) which participate in miRNA biogenesis 37. However, categorizing miRNAs inhibitors or drivers of tumorigenesis is sometimes not clear‐cut, since their activity depends upon the expression of their targets in the tissue/cell type in which they are expressed. The expression of certain miRNAs can be of prognostic value in human cancers 38. Furthermore, it was recently shown that miRNAs can be released through exosomes from cancer cells into body fluids including blood, urine, milk, sputum and saliva 39. Pharmaceutical approaches to the modulation of miRNA activities represent an exciting and promising field in cancer therapeutics 40, 41. In the following paragraphs we highlight some of the most prominent examples of ncRNAs with important roles in cancer.

Calin and associates were the first to describe a role of miRNAs in cancer, when they reported that miR‐15 and miR‐16 are dramatically downregulated in the majority (68%) of patients with B‐cell chronic lymphocytic leukaemia (CLL) due to deletions or mutations on the 13q13.4 chromosome 42. Both miR‐15 and miR‐16 induce apoptosis by repressing Bcl‐2, an antiapoptotic protein overexpressed in malignant nondividing B cells and many solid tumours 43. The New Zealand Black (NZB) mouse model of CLL exhibits genetic alterations in the mir‐15a/16‐1 locus, which results in decreased levels of miR‐15a and miR‐16 in lymphoid tissues 44, whereas the restoration of miR‐16 levels in a New Zealand Black–derived malignant B‐1 cell line mitigates the proliferation of malignant B1 cells 45.

More than 50% of human tumours carry loss of function mutations in the tumour suppressor protein TP53 (p53) 46. P53 drives transcription of the miR‐34 family, which activates apoptotic pathways 47. At the same time, miR‐34a promotes p53 expression by targeting the antiageing factor Sirtuin‐1 (SIRT1), a negative regulator of p53 48. Reduced expression of miR‐34 has been observed in many cancer types 49, including human gliomas, with concomitant increased expression of the target oncogenes c‐Met, Notch‐1/2 and cyclin‐dependent kinase 6 (CDK6). MiR‐34a was used in a ‘miRNA replacement therapy’ approach, where a chemically synthesized miRNA ‘mimic’ of miR‐34a and a lipid‐based delivery vehicle were used to block tumour growth in mouse models of nonsmall cell lung cancer (NSCLC) 50. Subsequently, a liposomal formulation of a miR‐34a mimic became the first miRNA to enter a phase I clinical study (http://clinicaltrials.gov/ct2/show/NCT01829971). It was given intravenously in patients with primary liver cancer or other selected solid tumors or hematologic malignancies. However, the trial was halted after immune‐related severe adverse events were reported in some of the patients. MiR‐26a is an example of a miRNA whose expression is lost in hepatocellular carcinoma (HCC). It regulates the cyclins D1 and D2, which control cell cycle arrest, as well as ULK1, a critical initiator of autophagy that promotes apoptosis 51. The administration of chemically synthesized miR‐26a in a mouse model of HCC results in inhibition of cancer cell proliferation, induction of tumour‐specific apoptosis, and a dramatic slow‐down in disease progression 52.

The let‐7 miRNAs represent a large family of miRNAs that plays an important role in stem cell division and cell differentiation 53. Let‐7 family members are downregulated in many types of cancer, including lung cancer, gastric tumours, colon cancer, melanoma, ovarian cancer and Burkitt's lymphoma 54. Let‐7 miRNAs target several oncogenes including K‐RAS, c‐Myc and HMGA2, and therefore are considered as tumour suppressors 53. The oncofoetal RBP Lin28 and its paralogue Lin28b bind to the terminal loops of most let‐7 precursors and block their processing into mature miRNAs 55, 56. Lin28 is a stem cell pluripotency factor and both paralogues are upregulated in many human cancers including glioblastoma, ovarian, gastric, prostate and breast cancer 37. The Lin28/let‐7 axis is not only prominent in cancer: it also regulates glucose metabolism through the let‐7‐mediated repression of multiple components of the insulin‐PI3K‐mTOR pathway 57. Aberrant glucose metabolism is tightly linked to cancer since a switch towards glycolytic metabolism increases the cancer cell's ability to increase biomass (‘Warburg Effect’). A subsequent study has shown that overexpression of either Lin28 or Lin28b in liver cancer cells elevates glucose uptake, lactate production and oxygen consumption, all of which are reversed upon addition of let‐7 mimics 58. The importance of the Lin28/let‐7 axis has spurred efforts to generate inhibitors of this biology with a new class of future anticancer agents 41. The oncogenic potential of Lin28 was also shown when King and associates constitutively expressed LIN28B in colon cancer cells and implanted them into immunocompromised mice. Tumours with constitutive LIN28B expression exhibited increased expression of colonic stem cell markers LGR5 and PROM1, mucinous differentiation and metastasis 59. Transgenic mouse models overexpressing Lin28B from the mouse Vil1 promoter specifically in the intestine, showed let‐7‐dependent intestine hypertrophy. Restoring mature let‐7a levels in the intestine reversed the observed hyperplasia, reducing the cellular transformation in the intestinal epithelium 60. Importantly, inhibition of either LIN28A or LIN28B *via* siRNAs suppressed established human xenograft tumours in mice 61. A similar effect was observed when the xenograft models were treated with chemically synthesized let‐7a miRNA.

Many miRNAs are found expressed at higher levels in tumours and can be seen as oncogenes. They promote tumour development by inhibiting tumour suppressor genes and/or genes that control cell cycle, cell differentiation and apoptosis. c‐Myc is an important oncogene that transactivates several miRNAs including the miR‐17~92 and miR‐106a~363 clusters 36. miR‐17~92 is a notable oncogenic miRNA cluster comprising six miRNAs that are located at chromosome 13q31, a genomic locus amplified in several types of lymphoma and solid tumours 62. This cluster is highly expressed in embryonic cells 63 and its miRNAs target the E2F transcription factor which controls the transition from G1 to S phase 64. The cluster is also overexpressed in many types of cancer, including B‐cell lymphoma, colon cancer, pancreatic cancer, breast cancer, ovarian cancer and neuroblastoma 65. MiRNAs from miR‐17~92 target Bim, repressing its proapoptotic activity 63 and the cell cycle inhibitors p21^CIP1^ and p57^KIP2^ thereby enhancing cancer cell growth 66, whereas miR‐19a and miR‐19b‐1 regulate the tumour suppressor PTEN 63. Xiao and associates generated mice with elevated miR‐17~92 expression in lymphocytes; these developed lymphoproliferative disease and autoimmunity and died prematurely 67.

MiR‐221 and miR‐222 (miR‐221/222) are two highly homologous miRNAs, which are significantly overexpressed in several types of human malignancies 68. For example, elevated expression of miR‐222 has been reported to contribute to pancreatic cancer invasion by targeting the tissue inhibitor of MMP‐2 (TIMP‐2) 69. In human glioma cells, miR‐221/222 inhibits cell apoptosis by targeting the proapoptotic gene PUMA 70. In breast cancer, overexpression of miR‐221/222 promotes epithelial‐to‐mesenchymal transition by negatively regulating the adiponectin receptor 1 71, as well as trichorhinophalangeal 1 (TRPS1) 72, leading to increased cell migration and invasion. PTEN, a prominent tumour suppressor gene, is a confirmed target of miR‐221/222 in the breast cancer cell line MCF‐7 73. MiR‐222 promotes tumour progression in HCC 74 and lentivirus‐mediated silencing of miR‐221 suppresses proliferation of liver cancer cells and growth of hepatoma xenografts *in vivo* 75.

There is considerable evidence that miR‐21 has oncogenic properties, being involved in regulatory pathways of proliferation, apoptosis and metastatic potential 76. Its targets include PTEN, as well as PDCD4, and BTG2, which play important roles in oncogenic processes 77. Furthermore, it is strongly upregulated in glioblastoma, head and neck carcinoma, ovarian cancer, B‐cell lymphoma and hepatocellular and cervical carcinoma 78. In a study of 540 clinical cancer samples by Volinia *et al*. 79, miR‐21 was the most consistently upregulated miRNA. Furthermore, mice conditionally expressing miR‐21 *via* Tet‐Off and Cre‐recombinase technologies developed clinical signs of haematological malignancies. MiR‐21‐overexpressing tumour cells were found to invade the peripheral blood, and other organs. Once miR‐21 expression was switched off, the tumours regressed, partly due to the activation of apoptosis 80.

On the list of prominent tumour‐promoting miRNAs is miR‐155, which originates from the B‐cell integration cluster, also known as MIR155HG (miR‐155 host gene). Aberrant expression of miR‐155 has oncogenic potential in several types of haematological malignancies 81. It was recently found that miR‐155 induces resistance to chemotherapeutic agents, which can be reversed by treatment with miR‐155 inhibitors, and that this chemoresistance is dependent on a p53/miR‐155 feedback loop 82. Eμ‐mmu‐miR155 transgenic mice express murine miR‐155 under the control of a VH promoter‐Ig heavy chain Eμ enhancer, which becomes activated at the pro‐B‐cell stage of B‐cell development. These mouse models develop a lymphoproliferative disease, which phenocopies the human form. This study was the first to demonstrate that transgenic overexpression of a single miRNA is sufficient to cause cancer 83.

Long noncoding RNAs have been shown to influence many of the pathways which drive malignant transformation. For instance the lncRNA MALAT1 (also known as NEAT2) is found to be highly expressed in many tumours 84, for example during metastasis in patients with early‐stage NSCLC 85. The elevated expression of MALAT1 is linked to traits such as increased migration, metastasis and clonogenic growth in NSCLC 85, pancreatic 86 and prostate cancer cells 87. Consistent with this, the deletion of MALAT1 in osteosarcoma cell lines inhibited cell proliferation and invasion 84. This lncRNA also promotes the growth and migration of ovarian cancer cells 88. It can bind to active chromatin sites 89 and it co‐localizes with nuclear speckles, where it influences pre‐mRNA splicing 26. MALAT1 is required for G1/S and mitotic progression by modulating the expression and/or pre‐mRNA processing of cell cycle–regulating transcription factors 90.

The Hox transcript antisense intergenic RNA known as HOTAIR is a lncRNA which is transcribed from the HOXC locus. It is considered a biomarker for the prognosis of certain cancers: higher levels of the RNA have been found in colorectal, liver, pancreatic, breast and gastric cancers 91. It forms double stem‐loop structures that bind to lysine‐specific demethylase 1 and PRC2 histone‐modification complexes, which leads to histone H3 tri‐methylation at lysine 27 (H3K27me3) and histone H3 dimethyl Lys4 (H3K4me2) and consequently results in gene silencing. HOTAIR is upregulated in breast cancer and increases cancer invasiveness and metastasis 92.

The lncRNA neuroblastoma associated transcript‐1 (NBAT‐1) was identified as an independent prognostic biomarker, predicting clinical outcome of neuroblastoma patients 93. Loss of NBAT‐1 increases cellular proliferation and invasion. It mediates epigenetic silencing of target genes, through its interaction with the PRC2 repressive chromatin complex.

The lncRNA ANRIL shows increased expression in NSCLC tissues, and this correlates with stages of tumour–node–metastasis and the size of tumours 94. ANRIL is expressed highly in gastric cancers, and higher levels of ANRIL promote proliferation of gastric cancer cells, where it inhibits apoptosis by epigenetic silencing of miR‐99a and miR‐449a transcription 95.

The oncofoetal lncRNA H19 is an important factor in both embryonic development and tumorigenesis. It is upregulated in a series of cancer types, where it reportedly accelerates cellular proliferation rates and increases the resistance of tumour cells to stress 96. Interestingly, H19 transcript has been reported to sequester and inhibit two cancer‐related miRNAs – let‐7 and miR‐106a 97, 98. H19 also serves as a primary miRNA precursor of miR‐675 99, which is considered as oncogenic due to its targeting of the tumour suppressor retinoblastoma protein. The H19 locus belongs to a cluster of imprinted genes that control embryonic and postnatal growth. The H19 gene is located 90 kb distant from the Igf2 gene on chromosome 11p15 in humans and chromosome 7 in mice. The Igf2 locus encodes insulin‐like growth factor‐2 (IGF2), which is a growth‐promoting peptide hormone highly expressed during embryogenesis. H19 and Igf2 genes are reciprocally imprinted from the maternal and paternal alleles respectively. The changes in imprinting of the Igf2‐H19 locus are likely to be involved in tumour formation. In humans, loss of imprinting at this locus are associated with the Beckwith–Wiedemann syndrome (BWS), which is characterized by overgrowth phenotypes in affected children, as well as a predisposition to develop embryonal tumours such as Wilms’ tumour or rhabdomyosarcomas 100. There are inconsistencies between various murine models which aim to define the role of H19 locus in cancer. In some cases, the H19 locus has been suggested to act as a tumour suppressor, and mice bearing a mutation in the Apc gene are murine models for colorectal cancer. When double mutants were generated, lacking both H19 and Apc, they showed an enhanced cancer phenotype compared with their Apc littermates 101. In other cases, H19 has been shown to promote tumour growth in mice. Matouk and associates demonstated that ectopic H19 expression enhances the tumorigenic potential of bladder carcinoma cells *in vivo* 102.

### MiRNAs and long noncoding RNAs in cardiovascular disease

Cardiovascular disease and complications thereof are a leading cause of morbidity and mortality worldwide. The myocardium can undergo remodelling in response to external stressors. However, chronic activation of remodelling processes, such as hypertrophy and fibrosis, can result in multiple cardiovascular diseases, including myocardial infarction, cardiomyopathies and heart failure. Ikeda *et al*. 103 identified significantly altered miRNA expression profiles in heart disease and showed that patterns of miRNA expression are distinct in different forms of heart disease. A myriad of studies has shown that miRNAs regulate the expression of genes in signalling pathways associated with heart failure, hypertrophy, and ischaemia reperfusion injury. For example, miRNAs have been found to promote or inhibit cardiomyocyte apoptosis, regulate postischaemic neovascularization and control cardiac fibrosis 104. Remarkably when miRNA biogenesis is inhibited through Dicer deletion, dilated cardiomyopathy associated with heart failure is observed in neonates 105, whereas the postnatal myocardium‐specific Dicer deletion drives maladaptive cardiac remodelling 106. Additionally, endothelial knockout of Dicer leads to endothelial dysfunction, revealing a key role for miRNAs in endothelial physiology 107.

Several miRNAs play key roles in vascular development and angiogenesis. For example miR‐24 has a role in cardiac vascularization 108. It is highly expressed in cardiac endothelial cells (ECs) and is significantly upregulated after cardiac ischaemia. Blockage of miR‐24 limits myocardial infarct size of mice, preventing endothelial apoptosis and enhancing vascularity. This miRNA exerts its functions through targeting the endothelium‐enriched transcription factor GATA2 and the p21‐activated kinase PAK4. MiR‐126‐3p is a proangiogenic factor, which is implicated in endothelial gene expression and mediates EC dysfunction as well as atherosclerosis triggered by blood flow changes 109. Overexpression of miR‐126‐3p reduces atherosclerosis 110, whereas its knock‐out causes systemic oedema, multifocal haemorrhages and ruptured blood vessels 111. It is enriched in the apoptotic bodies of dying ECs in a mouse model of atherosclerosis and has an angioprotective role *via* the CXCL12‐CXCR4 pathway 110.

MiR‐208 is selectively expressed in cardiomyocytes, and is highly expressed in autopsy samples of infarcted heart tissue from patients with myocardial ischaemia 112. In addition, compared to other miRNAs, levels of miR‐208 are high in cardiac tissue of dilated cardiomyopathy patients and it is a strong predictor of clinical outcome 113. In response to cardiac stress such as pressure overload, knockdown of miR‐208 in mice produces no cardiomyocyte hypertrophy and fibrosis 114. The miRNA also plays an important role in cardiac conduction, by regulating the expression of cardiac transcription factors and the gap junction protein connexin 40 (Cx43) 115. The miR‐15 family includes six closely‐related miRNAs that are also increased in myocardial ischaemia 116. Inhibition of miR‐15 family members by antimiR‐oligonucleotides reduces infarct size after ischaemia–reperfusion injury in cardiac tissue of both mice and pigs by de‐repressing the antiapoptotic protein Bcl‐2 and the mitochondrial protecting factor ADP‐ribosylation factor‐like protein 2 116.

Zidar and associates have reported that downregulation of miR‐150 is involved in the pathology of ventricular rupture after myocardial ischaemia 117. Of note, it was recently shown that the cardio‐related lncRNA ZFAS1 can interact directly with miR‐150, acting as a miRNA sponge that induces cardiomyocyte apoptosis in acute myocardial ischaemia *via* C‐reactive protein (CRP) 118. It regulates adenoreceptor beta 1 and CRP genes, which are associated with heart remodelling 119.

The neurologic‐enriched miRNA miR‐212/132 family becomes activated during heart failure 120. These miRNAs affect cardiac hypertrophy by targeting the anti‐hypertrophic and proautophagic transcription factor forkhead box O3 (FoxO3), leading to induction of the prohypertrophic calcineurin/NFAT signalling pathway 121. Altered levels of miR‐21 are associated with multiple cardiovascular diseases, including proliferative vascular disease, cardiac hypertrophy, heart failure, and ischaemic heart diseases 122. MiR‐21 promotes cardiac fibrosis by regulating genes, such as transforming growth factor β1 receptor III (TβRIII) 123 and matrix metalloprotease‐2 (MMP2) 124, 125. Bang and associates have demonstrated that miR‐21 is transferred through fibroblast derived exosomes, acting as a paracrine mediator of cardiomyocyte hypertrophy 125.

MiR‐1 is most abundantly expressed in heart and plays essential roles in cardiogenesis and in physiological cardiac function. Jayawardena *et al*. 126 showed that miR‐1 alone is sufficient to induce the fibroblast to cardiomyocyte reprogramming. MiR‐1 targets genes that cluster into several categories, including regulators of cell cycle, cardiac differentiation and the conductive system 127, 128. Cardiac Serca2a, which regulates calcium uptake into the sarcoplasmic reticulum (SR), has also been shown to increase after miR‐1 gene transfer in mice 129. This miRNA attenuates cardiomyocyte hypertrophy in cultured cardiomyocytes and in the intact adult heart by regulation of cardiomyocyte growth responses through modulation of calcium signalling components such as calmodulin 127. Consistent with this, miR‐1 has been found decreased in early‐stage cardiac hypertrophy 130. The miRNA and its primary target Errβ act together to regulate the transition from prenatal to neonatal stages by repressing the cardiac foetal gene program, which is reactivated under pathological conditions 131. Expression of miR‐1 is lost in the myocardium of myotonic dystrophy patients, concomitant with up regulation of its targets Connexin 43 (Cx43) and calcium voltage‐gated channel subunit alpha1C (CAV1.2) may at least partly account for the arrhythmia, which is observed in these patients 128. MiR‐1 is clustered together with miR‐133 on mouse chromosome 2, where they are separated by 9.3 kb, and on mouse chromosome 18, where they are separated by 2.5 kb 132. Although miR‐1 and miR‐133 derive from the same miRNA polycistron and are transcribed together, they have antagonistic effects on muscle development: miR‐1 enhances myogenic differentiation, whereas miR‐133 induces myoblast proliferation 133. MiRNA‐133 is decreased in mouse and human models of cardiac hypertrophy 134 through its regulation of the Ras homolog family member A (RhoA) and cell division control protein 42 homolog (Cdc42). It also plays a role in cardiac fibrosis by controlling the expression of the connective tissue growth factor 135. MiR‐133 affects inotropism by regulating the expression of multiple components of the β1‐adrenergic cascade, including the receptor itself 136.

Next to miRNAs, lncRNAs also play important roles in cardiovascular disease. In fact, data from deep sequencing demonstrated that compared to mRNA and miRNA expression profiles, lncRNA expression profiles are more sensitive to different heart failure aetiologies and that altered lncRNAs reflect increased susceptibility to coronary artery disease, myocardial infarction and heart failure 137. For example, Viereck *et al*. have recently discovered a new lncRNA – Chast (for ‘cardiac hypertrophy–associated transcript’) – that promotes cardiac remodelling and hypertrophy in mice. Antisense‐mediated degradation of Chast attenuated pathological cardiac remodelling, as it was shown by *in vivo* gain‐ and loss‐of‐function experiments in mice 138.

Besides its role in cancer, the lncRNA MALAT1 is also linked to cardiovascular disease: silencing of MALAT1 reduces capillary growth in a mouse model of hind limb ischaemia 139 as well as in a rat model of diabetic retinopathy 140. Furthermore, MALAT1‐derived mascRNA (MALAT1‐associated small cytoplasmic RNA) is involved in cardiovascular innate immunity and viral myocarditis 141.

The lncRNA GAS5 (growth arrest–specific 5) is another regulator of hypertension‐related vascular remodelling 142. It is mainly expressed in ECs and vascular smooth muscle cells (VSMCs), and its expression is significantly downregulated in hypertension. GAS5 regulates EC and vascular smooth muscle cell function through β‐catenin signalling. The cardiac apoptosis‐related lncRNA (CARL) has been found to regulate mitochondrial homeostasis and cell death in cardiomyocytes 143. CARL intervenes during the mitochondrial fission process by sequestering miR‐539 and inhibiting the miR‐539‐mediated repression of Prohibitin 143.

Ounzain *et al*. 144 identified several lncRNAs with potential roles in both cardiac development and pathological cardiac remodelling. One particular novel lncRNA, Novlnc6, is significantly decreased in dilated cardiomyopathy. Knockdown of Novlnc6 in cardiomyocytes results in a concomitant downregulation of BMP10 and NKX2.5, two important mediators of cardiac growth and function.

The lncRNA cardiac hypertrophy related factor, is substantially elevated in response to hypertrophic stimulation by angiotensin II in cardiomyocytes 145. In addition, it is also significantly upregulated in a mouse model of transverse aortic constriction and in human heart failure samples 145. This lncRNA acts as a sponge for miR‐489, de‐repressing Myd88, a direct target of miR‐489 so as to regulate cardiomyocyte hypertrophy.

Finally, the lncRNA myocardial infarction–associated transcript (MIAT) is highly expressed in heart and foetal brain tissue. Polymorphisms in MIAT that were identified by genome‐wide association studies are a risk factor for myocardial infarction 146. MIAT is found at low levels in platelets from patients with myocardial infarction 147, whereas elevated levels of this lncRNA are found in myocardial samples from patients with dilated cardiomyopathy suffering from Chagas disease 148.

### MiRNAs and long noncoding RNAs in neurodegenerative disease

Neurodegenerative diseases are hereditary and sporadic conditions which are characterized by progressive dysfunction and the death of neurons. According to the neuronal populations afflicted, these disorders can lead to disturbances in motor, cognitive and/or behavioural performance of affected individuals. They include diseases such as Alzheimer's disease (AD) and other dementias, Parkinson's disease (PD), amyotrophic lateral sclerosis (ALS), spinal muscular atrophy (SMA), Huntington's disease (HD) and others.

### MiRNAs in Parkinson's disease

MiRNAs display specific temporal and spatial patterns of expression during embryonic neural development and in adult brain 149. In the central nervous system, they have been shown to participate in a wide range of processes, such as neurodevelopment, brain architecture, neuroplasticity establishment, neurotransmission, etc. Not surprisingly, misregulated miRNAs have been linked to many neurodegenerative and psychiatric disorders. MiRNAs miR‐34b and miR‐34c are decreased in the affected areas of PD patients at an early stage of the disease 150. This miRNA family regulates alpha‐synuclein, a key protein in PD pathogenesis 150. Reduced expression of these miRNAs is associated with mitochondrial abnormalities and increased oxidative stress. MiR‐155 has been shown to mediate immune activation by aggregated α‐synuclein. In a PD mouse model overexpressing α‐SYN (*via* an adeno‐associated‐virus; AAV2‐SYN), levels of miR‐155 are significantly increased. However, miR‐155 knockout mice models transduced with AAV2‐SYN, exhibit a remarkably decreased proinflammatory response, without a loss of dopaminergic neurons 151. It was recently shown that miR‐30e improves neuronal damage, neuroinflammation and dyskinesia *via* targeting Nlrp3 expression and inhibiting NLRP3 inflammasome activation in a MPTP‐induced PD mice model 152. MiR‐30e levels were downregulated after MPTP injection, suggesting miR‐30 might also have a role in the pathogenesis of PD. MiR‐124 expression was downregulated in substantia nigra dopaminergic neurons following MPTP administration in mice. A MiR‐124 mimic delivered to the right lateral ventricle in the MPTP mouse model increases the density of tyrosine hydroxylase positive (TH+) neurons and reduced the upregulation of Bim mRNA level and protein level induced by MPTP, leading to reduced apoptosis 153.

### MiRNAs in Alzheimer's disease

Expression of the miR‐29a/b‐1 cluster is significantly decreased in the brains of patients suffering from sporadic AD, displaying abnormally high levels of BACE1 protein 154. This miRNA family targets BACE‐1 secretase, which cleaves amyloid precursor protein (APP) and generates toxic Aβ species, thereby contributing to synaptic loss and cognitive decline in AD. MiR‐29 has been also suggested to protect cells from apoptosis by targeting proapoptotic proteins, including BIM, BMF, HRK and PUMA 155. It was recently demonstrated that pre‐miR‐29b encapsulated in polyplexes decreases levels of hBACE1 and Aβ45.^156^ Levels of miR‐29a are increased by more than two‐fold in cerebrospinal fluid of AD patients, indicating that miR‐29a may be a candidate biomarker for AD 157. MiR‐106b from the miR‐106b~25 cluster is a regulator of Aβ production and clearance through the suppression of ABCA1 expression 158. Suppression of ABCA1 expression by miR‐106b impairs cellular cholesterol efflux and increases the levels of secreted Aβ. MiR‐106b is also aberrantly expressed in a double transgenic mouse model for AD 159. Simvastatin was recently shown to ameliorate the memory decline in AD mouse models *via* decreased miR‐106b levels 160. Finally, miR‐34a is found over expressed in affected brain regions of AD patients as well as in transgenic AD mice 161. The increased expression of miR‐34a in specific brain regions induces synaptic dysfunction. Its accumulation, along with the interneuronal transfer of miR‐34a‐loaded exosomes, may affect neural networks dedicated to memory. MiR‐34c is also connected to hippocampal memory function. Inhibition of this miRNA rescues memory impairment in AD transgenic mice, with concomitant de‐repression of SIRT1, a confirmed target of miR‐34 162.

### MiR‐196 in Huntington's disease

Huntington's disease is an autosomal‐dominant disease that is caused by an expansion of CAG trinucleotide repeats located in the exon 1 region of the huntingtin gene. MiR‐196a has emerged as a protective miRNA in the context of HD. Overexpression of miR‐196a leads to a reduction of mutant huntingtin (HTT) and the formation of pathological aggregates in HD models of human embryonic kidney cells and mouse neuroblastoma cells. In HD transgenic mice overexpressing miR‐196a, suppression of mutant HTT in the brain shows attenuated neuropathological progression, manifested by reduced nuclear, intranuclear and neuropil aggregates as well as late‐stage behavioural phenotypes 163. The effects of miR‐196a might be *via* its involvement in the ubiquitin–proteasome systems, gliosis, and the CREB pathway.

### MiR‐183 in spinal muscular atrophy

MiR‐183 has been shown to contribute to the pathology of SMA *via* its target mTor. The local axonal translation of mTor is reduced in SMN‐deficient neurons, and this can be restored by inhibition of miR‐183. Importantly, inhibition of miR‐183 expression in the spinal cord of an SMA mouse model prolongs survival and ameliorates motor performance of SMN‐mutant mice 164.

### BACE1‐AS in Alzheimer’ s disease

Aberrant lncRNA expression is linked to the onset and progression of several neurodegenerative diseases. For instance BACE1‐AS is a NAT that is transcribed from an intron of the β‐secretase‐1 (BACE1) gene in antisense direction. Its expression is elevated in subjects suffering from AD and in APP transgenic mice 165. Several cell stressors increase BACE1‐AS RNA, which enhances BACE1 mRNA stability, generating additional Abeta 1‐42. It has been postulated that BACE1‐AS prevents translational repression of BACE1 mRNA by miR‐485, by masking the miRNA binding site 31.

### MALAT1 and NEAT1_2 in FTLD and ALS and Huntington's disease

TDP‐43 is a nuclear RNA‐binding protein that forms inclusion bodies in frontotemporal lobar degeneration (FTLD) and ALS. The binding of TDP‐43 to MALAT1 and NEAT1_2 lncRNAs is increased in human FTLD brains compared with healthy controls 166. Analyses of human spinal motor neurons in ALS cases shows that NEAT1_2 lncRNA is upregulated during the early stage of ALS pathogenesis. This lncRNA acts as a scaffold for RNAs and RBPs in the nuclei of ALS motor neurons, thereby modulating the functions of ALS‐associated RNA‐binding proteins, such as TDP‐43 and FUS/TLS, during the early phase of ALS 167. NEAT1 levels are also increased in the postmortem brain from patients of HD 168. Gain‐of‐function studies showed that NEAT1 upregulation in HD contributes to the neuroprotective mechanism against neuronal injury.

### UCHL1‐AS, MALAT‐1 and HOTAIR in Parkinson's disease

The ubiquitin carboxy‐terminal hydrolase L1 gene (UCHL1) is closely related to brain function and neurodegenerative diseases. An antisense transcript of UCHL1, UCHL1‐AS promotes translation of UCHL1 169, which is strongly attenuated in neurochemical models of PD *in vitro* and *in vivo* 169. MALAT1 is highly expressed in neurons 170. It was recently demonstrated that MALAT1 overexpression increases, whereas inhibition decreases alpha‐synuclein expression 171. β‐Asarone, a constituent of Acorus tatarinowii Schott, suppresses the levels of MALAT1 and alpha‐synuclein in the midbrain tissue of PD mice, suggesting that β‐asarone may be a potential therapeutic agent for PD^171^. HOTAIR is upregulated in a mouse model of PD that is produced by intraperitoneal injection of MPTP, a prodrug to the neurotoxin MPP+. The lncRNA increases the stability of LRRK2 mRNA 172, and thus may interfere with the LRRK2‐associated mitochondrial impairment in PD.

## Conclusion

The constellations of physiological processes which orchestrate life are subject to intricate control. MiRNAs and lncRNAs have emerged as ubiquitous RNA molecules capable of modulating all cellular processes. In particular, ncRNAs have drawn great attention partly for their putative roles in the pathology of many diseases. In many of the cases highlighted in this Review, in which we have limited the discussion to three types of diseases, the links between ncRNAs and disease pathologies came to light through their aberrant expression in disease cells or tissues. It is noteworthy that some ncRNAs (e.g. miR‐15, miR‐29, miR‐34, ANRIL and MALAT‐1) appear to contribute to more than one pathological mechanisms. In some of these cases, the miRNA‐disease association is sufficiently strong (i.e. possibly causative) that the miRNA represents a potential drug target or a therapeutic entity (e.g. miR‐106 and let‐7 respectively). The availability of potent pharmacological tools for use in animal models of these diseases and/or in clinical trials will ultimately clarify their value in distinct therapeutic applications 173.
